# Evaluation of the effect of age of the younger mice on the rejuvenation of the older mice by heterochronic parabiosis

**DOI:** 10.18632/aging.203966

**Published:** 2022-03-21

**Authors:** Yushi Suzuki, Kento Takaya, Shiho Watanabe, Marika Otaki, Hikaru Kono, Kazuo Kishi

**Affiliations:** 1 Department of Plastic and Reconstructive Surgery, Keio University School of Medicine, Shinjuku-ku, Tokyo 160-8582, Japan

**Keywords:** senescence-associated secretory phenotype, heterochronic parabiosis, rejuvenating effect, inflammatory cytokines, aging

## Abstract

Heterochronic parabiosis is used to study the systemic effects of aging and involves surgically connecting two animals of different ages such that they have common blood circulation. Although this technique has been prevalent for a long time, there is no scientific consensus on the age of the animals that should be used. We hypothesized that the younger the animal, the greater would be its rejuvenating effect. Hence, to test this hypothesis, we created parabiosis of 67-week-old mice with younger mice of different ages (4-week-old and 8-week-old). We evaluated the changes in appearance and the expression IL-1A, IL-6, and Cdkn2a (p16) in the liver, kidney, brain, and skin. These cytokines belong to the senescence-associated secretory phenotype (SASP) factors, and are indicators of aging. Although we did not find any significant changes in the appearance of the mice, we found statistically significant differences in some SASP factors between the liver of the 4-week-old and 8-week-old pairs. However, overall, compared to the 8-week-old mice, the 4-week-old does not exert a significantly higher rejuvenation effect on the older mice. Hence, we concluded that the rejuvenation of older mice during heterochronic parabiosis might not be affected by the exact age of the younger mice.

## INTRODUCTION

Parabiosis is an experimental technique in which two animals are surgically joined to bring about a common blood circulation. This research method has been around since the 1800s [1], and it helps to understand the effect of the systemic factors of one animal on the other. Heterochronic parabiosis is a variant of this technique, in which we join an older animal with a younger one. This technique examines the effects of the systemic factors of the younger animal on the aging process of the older animal, including cell and tissue aging, the onset of age-related diseases, and the organism’s lifespan. This experimental method was published in the late 1950s and the early 1960s, and several studies have looked into it due to the claim that heterochronic parabiosis might extend an organism’s lifespan [2–4].

In recent years, many aging studies have conducted heterochronic parabiosis on mice. However, there is no unified view on the age of mice to be used in these kinds of experiments. For example, the age of the younger mice ranges from 2 to 6 months in several studies [5–9]. We hypothesized that the younger the animal, the greater would be its rejuvenating effect on the older animal. Hence, we created heterochronic parabiosis of older mice with younger mice of different ages to test this hypothesis. We evaluated this rejuvenating effect by observing the changes in the appearance of mice and by examining the secretion of pro-inflammatory cytokines called senescence-associated secretory phenotype (SASP) factors as indicators of aging.

## RESULTS

### Evaluation of the appearance of the older mice

**Figure 1:**
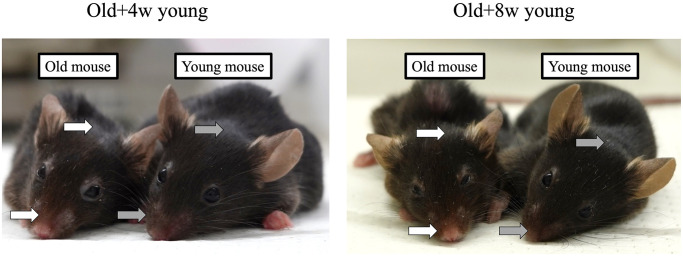
**A 67-week-old mouse was joined to a 4- or 8-week-old mouse.** Although the mice pairs were joined for 2 months, the fur around the nose and head of the older mice (white arrows) was sparser than that of the younger mice (grey arrows), and no apparent rejuvenation was achieved in either case after 2 months.

The characteristic appearances of older mice not present in younger mice include sparse fur and peeling around the nose. We did not observe any improvement in these characteristics in the older mice after 2 months of parabiosis, regardless of whether they were joined with 4-week-old or 8-week-old mice (Figure 1).

### Evaluation of SASP factors

**Figure 2:**
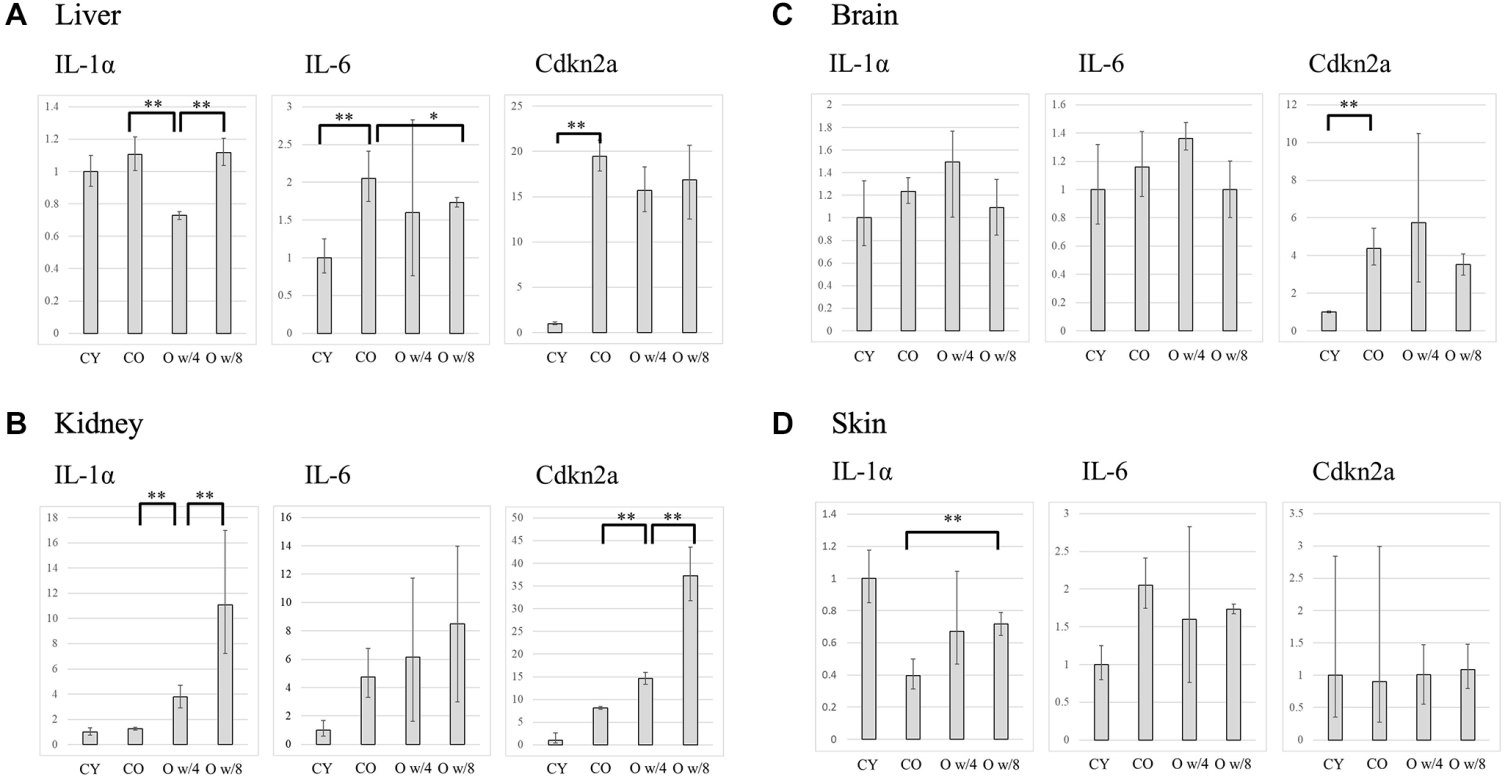
Expression levels of IL-1α, IL-6, and Cdkn2a in the liver (**A**), kidney (**B**), brain (**C**), and skin (**D**) of the control and parabiosis groups. Abbreviations: CY: control young; CO: control old; O w/4: 67-week-old mice joined with 4-week-old mice; O w/8: 67-week-old mice joined with 8-week-old mice. ^*^P < 0.05, ^**^P < 0.01.

**Figure 3:**
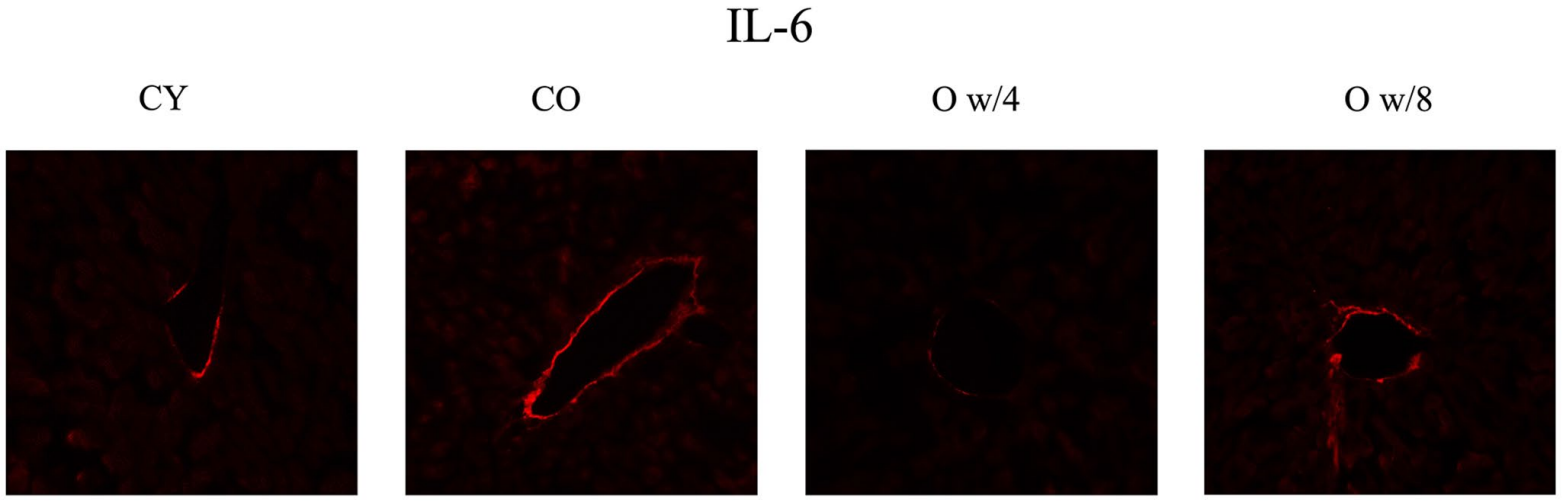
**Immunofluorescence staining of the liver.** Red signifies IL-6. IL-6 expression is observed along the wall of the interlobular vein. The 67-week-old mice joined with 4-week-old mice have decreased fluorescence compared to the 67-week-old mice joined with 8-week mice, suggesting that there is an effect of parabiosis on younger individuals. Abbreviations: CY: control young; CO: control old; O w/4: 67-week-old mice joined with 4-week-old mice; O w/8: 67-week-old mice joined with 8-week-old mice.

Next, we compared the expression levels of the SASP factors IL-1A, IL-6, and Cdkn2a (p16) in the liver, kidney, brain, and skin of the conjoined mice pairs. In the liver, there was no significant difference in gene expression between the young and old control mice. However, the expression of *Il-1a* and *Il-6* was significantly lower in the older mice joined to the 4-week-old mice than the one joined to the 8-week-old mice (Figure 2A). This result was also corroborated by the immunofluorescence data on IL-6 expression in the liver (Figure 3). Although the expression of Cdkn2a was significantly higher in the old control mice than in the young control mice, we did not find any statistically significant rejuvenating effect in the parabiosis mice groups (Figure 2A).

In the kidneys, heterochronic parabiosis did not show any rejuvenating effect, that is, the gene expression of the inflammatory cytokines was not suppressed. In contrast, the expression of each gene increased in the 67-week-old mice by parabiosis, especially in the connection with the 8-week-old mice (Figure 2B).

In the brain, the expression of each gene increased in the older mice paired with the 4-week-old mice than in the control groups, although the difference was not significant. No significant changes were observed between the 67-week-old mice of the two parabiosis groups (Figure 2C).

As for the skin, there was a large variation in gene expression among the groups. However, there were no statistically significant changes in gene expression between the young and old control groups. Further, we did not observe any statistically significant suppression of the SASP factors in the parabiosis groups (Figure 2D).

## DISCUSSION

The SASP factors included various inflammatory cytokines, chemokines, and extracellular matrix-degrading enzymes secreted by senescent cells, mainly by the activation of NF-κB [10, 11]. IL-1α and IL-6 are major SASP factors that act on surrounding cells to induce cellular senescence [12–14]. Further, DNA damage occurs during cellular senescence, activating the DNA damage response and triggering cell cycle arrest. p16, which is encoded by Cdkn2a, is strongly involved in this mitotic arrest pathway, and its expression increases with aging [15–17]. Hence, in this study, when we performed heterochronic parabiosis to rejuvenate old mice by connecting them with younger mice, we examined the changes in expression of the SASP factors IL-1a, IL-6, and Cdkn2a.

As described in the introduction, to test our hypothesis that the younger the animal, the greater is the rejuvenating effect, we created heterochronic parabiosis of older mice with younger ones of different ages.

The age of older mice was 67 weeks; these were connected with either 4-week-old or 8-week-old mice and the rejuvenation effect was evaluated. The ages of mice in the different groups were selected based on the considerations mentioned below. For heterochronic parabiosis, generally, the age of mice in the younger group is 8 weeks (2 months). We also set up a group of more younger mice, aged 4 weeks, in view of the fact that at this age, mice are completely weaned and start their advance toward sexual maturity. For the older group, 67-week-old mice were selected because mice of this age have completed the middle-age period of their lives and are approaching old age.

In the liver, although we found significant differences in some SASP factors between the 67-week-old mice joined to the 4-week and 8-week-old mice, we could not obtain evidence that the older mice joined to the 4-week-old animals showed a greater rejuvenation in general. Further, in the skin, there was no clear difference in the factors even between the old and young control mice. Hence, the effect of SASP factors is not uniform across all organs, and their effect on skin aging has not yet been clearly shown [18].

The limitations of this study include the fact that the control groups did not undergo parabiosis, so the effect of inflammation caused by surgical invasion could not be excluded. In addition, since the index of aging is not limited to SASP, and the factors that fall under SASP factors are not limited to the three factors used in this study and the analytic strategy is small, we cannot be sure that connection of the older mice with the 4-week-old mice did not affect rejuvenation. Hence, we would like to conduct further studies in the future. However, we believe that even after considering all the limitations of our study, the difference in the heterochronic parabiosis-induced rejuvenation effect of the 4-week-old and 8-week-old mice on the old mice may not be strong.

## MATERIALS AND METHODS

### Mice

C57BL/6JJmsSlc (Japan SLC, Inc.) mice (female) were used in this study. We obtained five pairs of mice: three pairs of 4-week-old mice and 67-week-old mice, and two pairs of 8-week-old mice and 67-week-old mice. As a control group, we took 12-week-old and 75-week-old mice that were not joined. This experiment was approved by Keio University Institutional Animal Care and Use Committee (4596).

### Preparation of heterochronic parabiosis

Parabiosis was performed according to the method of Kamran et al. [19]. One young and one old mouse were incised in the lateral abdomen. The elbow and knee joints of both mice were tightly fixed with nylon thread to prevent the joint from separating due to the movement of each individual. The skin on both sides of the lateral abdomen was then sutured tightly to firmly adhere the skin of the mice pair.

### Analysis method

Mice were sacrificed 2 months after parabiosis. The liver, skin, kidney, and brain were collected immediately after euthanizing the mice by cervical dislocation.

### Reverse transcription-polymerase chain reaction

Total RNA was extracted from tissues using ISOGEN reagent (Nippon Gene Co., Ltd., Japan). Gene expression was quantified using primers against *Il-6, Il-1a,* and *p16* and the PCR master mix (Cat. No. 4352042; Applied Biosystems, Foster City, CA, USA), according to the manufacturer’s instructions. *Actb* was used as the control gene for normalization. The level of gene expression in the untreated young group was used as the baseline, and fold change in gene expression was defined by the 2^−ΔΔCT^ method.

### Immunofluorescence

After fixation in 4% paraformaldehyde overnight, the liver sections were placed in 30% sucrose for 48 h. The frozen sections were then cut with a sliding microtome (10 μm) and stored in a cryoprotectant (OCT compound; Sakura Finetek Japan Co. Ltd., Japan) at −80^o^C until use. Immunostaining was performed using primary antibody against IL-6 (D5W4V, Cell Signaling Technologies, Danvers, MA, USA) and the Cy™3 AffiniPure Donkey Anti-Rabbit secondary antibody (Code: 711-165-152, Jackson ImmunoResearch Inc. West Grove, PA, USA).

### Statistical analysis

A paired *t*-test was performed to compare the gene expression levels in each group. The level of statistical significance was set at *P* < 0.05. All statistical analyses were performed using SPSS version 26 (IBM^®^).
